# Prevalence of *Staphylococcus aureus* colonization in patients for total joint arthroplasty in South Africa

**DOI:** 10.1186/s13018-020-01635-4

**Published:** 2020-03-31

**Authors:** Jurek Rafal Tomasz Pietrzak, Zia Maharaj, Lipalo Mokete

**Affiliations:** Charlotte Maxeke Johannesburg Academic Hospital, University of the Witwatersrand, Jubilee Street, Parktown, Johannesburg, Gauteng 2193 South Africa

## Abstract

**Background:**

Periprosthetic joint infections (PJIs) are a major source of morbidity and mortality for patients undergoing total joint arthroplasty (TJA). *Staphylococcus aureus* (*S. aureus*) colonization is an independent, modifiable risk factor for periprosthetic joint infections. Post-operative infections are reported to be ten times greater in *S. aureus* carriers than in non-carriers in developed countries though recorded data is lacking for the developing world. This study aims to determine the prevalence of *S. aureus* colonization in patients awaiting TJA in South Africa.

**Methods:**

We prospectively assessed 119 patients awaiting total knee arthroplasty and total hip arthroplasty between May and October 2016. We screened three separate anatomical sites on each patient for *S. aureus*. Patients with positive cultures were treated with intranasal mupirocin ointment and chlorhexidine body wash. Univariate and comparative statistical analyses to determine risk factors for colonization was conducted using *t* tests, Fisher’s exact tests, and chi-square analyses.

**Results:**

The overall prevalence of methicillin-sensitive *S. aureus* colonization was 31.9% (*n* = 38). There were no patients colonized with methicillin-resistant *S. aureus*. Nasal swabs returned a yield of 81.6% (*n* = 31), with groin swabs and axillary swabs at 39.5% (*n* = 15) and 28.9% (*n* = 11), respectively. Eradication was successful in 94.74% (*n* = 36) after 5 days treatment. All patients (100%) were decolonized after counseling and repeat eradication treatment. The overall complication rate was 7.6% (*n* = 9). The 30-day readmission rate in the *S. aureus*-colonized group was 7.9% (*n* = 3) as opposed to 7.4% (*n* = 6) in the non-colonized cohort. There were no 60- and 90-day readmissions and no cases were revised at a mean follow-up of 2.26 years.

**Conclusions:**

The rate of *S. aureus* colonization in patients undergoing elective TJA in a developing country was 31.9% and is equivalent to reported rates in developed countries. Eradication treatment with combined intranasal mupirocin ointment and chlorhexidine body wash is a successful treatment modality. A larger cohort of patients is recommended to determine risk factors and post-operative septic sequelae in this population group.

## Introduction

Total joint arthroplasty (TJA) is a commonly performed orthopedic operation and remains a reliable, reproducible, and cost-effective surgical procedure [1]. The demand for total hip arthroplasty (THA) and total knee arthroplasty (TKA) is high and continues to rise due to overall patient satisfaction [1]. The average rate of THA and TKA worldwide has increased by an estimated 30% and 100% respectively, between 2000 and 2015 [1]. Approximately 93,000 THA and 100,000 TKA procedures were performed in the United Kingdom (UK) in 2018 [2]. There were over 1 million TJA cases performed in the United States of America (USA) in 2018 [3], and this is projected to increase at an alarming rate to over 4 million annual procedures by 2030 [4]. This overwhelming demand represents a massive economic burden on healthcare systems which is compounded by readmissions up to 2 years post-operatively [5]. Approximately half of these readmissions are due to medical co-morbidities and unrelated to the surgical procedure itself [5].

Periprosthetic joint infections (PJI), including surgical site infections (SSI), are infective post-operative complications resulting in severe morbidity and mortality [6]. The cumulative incidence of PJI after primary THA and TKA is 1.4% after 10 years [7] and has a 5-year mortality rate of 21.12% [6]. PJI is the third most common cause of THA revisions and the most common cause of TKA revisions, worldwide [7]. In the USA, over 50% of 90-day readmissions after TKA are as a result of PJI [3]. The annual cost for revisions due to PJI was US$566 million in 2009 and is expected to increase to US$1.62 billion by 2020 [7]. Literature describes a well-established increased risk for PJI after TJA in patients colonized with *Staphylococcus aureus* (*S. aureus*) on their nasal on skin flora [8–10]. Furthermore, SSIs resulting from *S. aureus* colonization are higher in patients undergoing TJA when compared to other orthopedic procedures [11] and account for more than 60% of infections complicating THA or TKA [12].

The most common site of *S. aureus* colonization is the anterior nares but it can also be found asymptomatically on the skin of up to one third of the general population [13]. Nasal carriers of *S. aureus* are up to nine times more likely to develop a SSI than non-carriers [14]. The two primary subtypes of *S. aureus* are namely methicillin-sensitive *S. aureus* (MSSA) and the more virulent methicillin-resistant *S. aureus* (MRSA) [15]. The risk of infection in MRSA-colonized patients is greater than in patients that are not colonized with MRSA [15]. Furthermore, the length of hospital stay as a result of MRSA-related SSI is almost double in comparison with non-MRSA SSIs [15]. There is a geographical discrepancy with the incidence of *S. aureus* nasal colonization reported as ranging from 1.1 to 25% [16, 17]. The incidence of SSIs caused by *S. aureus* in the USA between the early 1990s until 2010 has increased from 20 to 30.4% [12]. In Africa, an associated additional risk factor for *S. aureus* colonization is concurrent human immunodeficiency virus (HIV) infection [18]. South Africa bears the greatest burden of HIV worldwide [19]; however, *S. aureus* surveillance data from African countries is scanty [20].

Pre-operative colonization with *S. aureus* is a recognized independent modifiable risk for infective complications after TJA [21]. Several studies have documented the prevalence of *S. aureus* in patients for TJA in developed countries; however, there are limited epidemiology reports of trends in the developing world [16, 17]. The aim of the study was to assess the prevalence of *S. aureus* colonization in patients admitted to a South African academic hospital for elective primary TJA and the subsequent incidence of PJIs in these patients.

## Methods

The study was a prospective analysis conducted in the Arthroplasty Unit at Charlotte Maxeke Johannesburg Academic Hospital in Gauteng between May and October 2016. A mathematical formula was used to calculate the sample size required for a prevalence study to achieve a statistical level of confidence of 95% [22]. The sample size required was calculated to be 100 subjects given an estimated regional prevalence rate of 30% [22, 23]. Patients included in the study were adults 18 years of age or older and undergoing elective primary THA or TKA. Exclusion criteria applied to patients unwilling to participate in the study or undergo swabbing for *S. aureus* pre-operatively. Additional exclusion criteria included patients presenting with traumatic neck of femur fractures for emergency primary THA, those who were unable to sign consent for the elective surgical procedure and/or participation in this study and/or for swabbing for *S. aureus* culture testing. Lastly, patients who required revision THA or TKA were excluded.

Medical clearance was obtained from the institutional ethics committee registered with the national ethics council of the Department of Health. Informed consent was obtained from all the eligible patients who were willing to participate in the study. These patients were assessed pre-operatively and demographic data was recorded including age, gender, body mass index (BMI), and tobacco use. Co-morbid conditions were also identified such as diabetes mellitus, rheumatoid arthritis, and HIV infection. Patients with unknown HIV status were consented for HIV screening and subsequently tested. The CD4+ T cell count (CD4+) and viral load (VL) of all HIV-positive patients were measured.

Three separate swabs of the anterior nares, axilla, and inguinal areas for each individual patient were sampled, before their planned operative procedures. Testing multiple sites for *S. aureus* colonization increases the likelihood of identifying potential carriers [12]. All samples were sent to the Infection Control Services Laboratory at the National Health Laboratory Services. Swabs were prepared on plates and after 18–24 h incubation, cultures with staphylococci-resembling colonies were picked off and these isolates got manual senses performed [24]. Results were ready within 72 h of sample collection and included identification results for MSSA and MRSA subtypes.

If the swabs did not culture *S. aureus* from any of the sites, those patients underwent TJA. Patients with positive cultures were provided with mupirocin ointment and two bottles of chlorhexidine wash. Several potential decolonization strategies exist with the most consistently successful eradication protocol involving the use of intranasal mupirocin ointment [25, 26]. Chlorhexidine body wash is used as an adjunct to mupirocin ointment [25, 26]. These patients were instructed to spray topical mupirocin intra-nasally twice per day and to apply chlorhexidine directly to the skin of their hips, knees, axilla, and groin with circular movements twice daily for 5 days.

After 5 days of treatment, the participants were subsequently re-swabbed and tested for *S. aureus* via the same process as detailed previously. Results were reviewed and those with negative cultures then received TJA. Patients who remained culture positive received repeat counseling and were placed on the eradication protocol again. They once again returned after five subsequent days and underwent re-swabbing and re-testing of all three areas. In the event that any of the third set of swabs remained positive for MSSA or MRSA, the patients would then be were referred to the Infectious Diseases Unit for further management.

All patients were monitored post-operatively after discharge with initial review after 17 days for removal of surgical incision clips. Participants were closely followed up post-operatively at 30 days, 6 weeks, 6 months, 1 year, and annually thereafter. They were, however, counseled and requested to return if there were any surgical incision concerns, wound ooze, or drainage before then. Patients were observed for a minimum follow-up period of 2 years and all complications were recorded.

Univariate and comparative statistical analyses were conducted to determine which variables were risk factors for *S. aureus* colonization by using independent samples *t* tests, Fisher’s exact tests, and chi-square analyses. Odds ratios and corresponding 95% confidence intervals were estimated using binary logistic regression analyses for *S. aureus* carriage for each potential risk factor. Statistical significance was set at *p* < 0.05.

## Results

There were 119 patients included in the study that were subdivided into 41 patients (34.5%) for THA and 78 patients (65.5%) for TKA. There were no patients colonized with MRSA, and the overall TJA prevalence of MSSA-colonization was 31.9% (*n* = 38) (Table 1). The *S. aureus* colonization rate for patients undergoing TKA (*n* = 26) and THA (*n* = 12) was 21.8% and 10.1% respectively; however, the difference was not statistically significant (*p* value = 0.182). Patients awaiting TKA were 1.7 times more likely to have *S. aureus* colonization than those awaiting THA (OR TKA = 0.58, OR THA = 0.33).

**Table 1:** Swab culture results of TJA population and anatomical sites

**Demographic**	**Sample (*n*)**	***S. aureus*prevalence**		***p*value**
		**%**	**95% CI (%)**	
**Total**	**119**			
TJA	38	31.9	24.1–40.7	0.067
TKA	26	21.8	13.2–30.1	
THA	12	10.1	3.7–21.2	
**Culture-positive samples**	**38**			
Nasal	31	81.6	67.2–91.4	0.000
Groin	15	39.5	25.2–55.3	
Axilla	11	28.9	16.5–44.5	

The anatomical sites screened returned yields of 81.6% (*n* = 31) for nasal swabs, 39.5% (*n* = 15) for groin swabs, and 28.9% (*n* = 11) for axillary swabs. Further inspection of the results (Table 2) revealed that of the 38 patients with *S. aureus* colonization, 42.1% (*n* = 16) tested positive in the anterior nares only, compared to 7.9% groin only (*n* = 3) and 5.3% axilla only (*n* = 2). There were no statistically significant risk factors identified according to demographic factors and co-morbidities (Table 3).

**Table 2:** Summary of *S. aureus* prevalence Sensitivity according to Anatomical Sites Screened

**Site**	***S aureus*prevalence**			***p*value**
	***n***	**%**	**95% CI (%)**	
Nasal only	16	42.1	27.5–57.9	0.000
Groin only	3	7.9	2.3–19.6	
Axilla only	2	5.3	1.1–15.8	
Nasal and groin	19	50	37.4–82.2	0.000
Nasal and axilla	18	47.3	40.9–65.1	0.000
Groin and axilla	5	13.2	5.4–18.2	0.333

**Table 3:** Prevalence of *S. aureus* colonization by demographic and co-morbid factors

**Demographic**		**Sample**	***S. aureus*prevalence**			***p*value**
	**Group**	***n***	***n***	**%**	**95% CI (%)**	
**Total sample**		**119**	**38**	**31.9**	**(24.1–40.7)**	
Age	< 50 years	10	3	30.0	(9.3–60.6)	0.639
	50–70	81	28	34.6	(24.9–45.3)	
	> 70 years	28	7	25.0	(11.9–42.9)	
Sex	Male	30	9	30.0	(16.0–47.7)	0.793
	Female	89	29	32.6	(23.5–42.8)	
Race	Black	79	26	32.9	(23.3–43.7)	0.877
	Colored	6	2	33.3	(7.7–71.4)	
	Indian	6	1	16.7	(1.9–55.8)	
	White	28	9	32.1	(17.2–50.5)	
Domicile	Rural	8	3	37.5	(11.9–70.5)	0.727
	Urban	111	35	31.5	(23.4–40.6)	
BMI (kg/m^2^)	< 30	44	17	38.6	(25.3–53.4)	0.484
	30–39	53	15	28.3	(17.6–41.3)	
	> 40	22	6	27.3	(12.3–47.8)	
Previous TJA	Yes	38	15	39.5	(25.2–55.3)	0.089
	No	81	23	28.4	(19.5–38.8)	
Smoking	Yes	95	30	31.6	(22.9–41.4)	0.869
	No	24	8	33.3	(17.2–53.2)	
Alcohol	Yes	93	30	32.3	(23.4–42.2)	0.886
	No	26	8	30.8	(15.8–49.8)	
HIV	Positive	25	10	42.1	(22.3–64.1)	0.065
	Negative	94	28	30	(21.7–39.5)	
Diabetes	Yes	19	6	32	(14.4–53.9)	0.971
	No	100	32	31.6	(23.5–41.6)	
RA	Yes	102	29	28.4	(20.4–37.7)	0.45
	No	17	9	52.9	(30.3–74.6)	
Hypertension	Yes	43	15	34.9	(22–49.7)	0.604
	No	76	23	30.3	(20.8–41.2)	

Eradication was successful in 94.74% (*n* = 36) after 5 days treatment. There were two patients (5.26%) who failed eradication; however, all patients (100%) were decolonized after counseling and repeat eradication treatment.

**Fig. 1:**
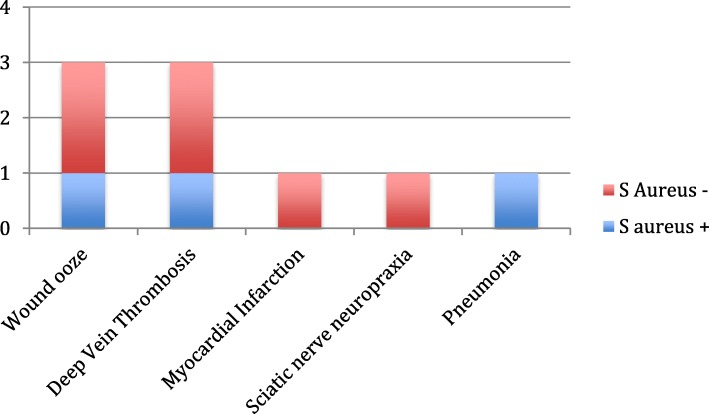
Cumulative 30-, 60-, and 90-day readmission rates and pathological causes

The overall complication rate was 7.6% (*n* = 9) (Table 4). There was no significant difference (*p* = 0.925) in the incidence of complications in those with or without *S. aureus* colonization. The 30-day readmission rate in the MSSA-colonized group was 7.9% (*n* = 3) as opposed to 7.4% (*n* = 6) in the non-colonized cohort. There were no 60- and 90-day readmissions. The causes for readmissions are depicted in Fig. 1. All three wound oozes occurred in patients after TKA. These were managed by readmission, withholding anti-coagulation for 2 days and icing the affected limb. The wound ooze in all three patients had stopped before discharge. All four readmissions were due to medical reasons, namely three deep vein thrombosis events and one myocardial infarction. There were no incidences of SSIs or PJIs in this cohort of patients. There were no cases of revision TJA for any cause in any patient at a mean follow-up of 37.34 months.

**Table 4:** Post-operative complications of *S. aureus*-colonized vs. non-colonized patients

**Pathological cause**	**Prevalence (%)**		
	**Total (*n*= 119)**	***S. aureus*+ (*n*= 38)**	***S. aureus*− (*n*= 81)**
All cause	7.6	7.9	7.4
Wound ooze	2.5	2.6	2.5
Deep vein thrombosis	2.5	2.6	2.5
Myocardial infarction	0.84	0	1.2
Sciatic nerve neuropraxia	0.84	0	1.2
Pneumonia	0.84	2.6	0

*p* value = 0.925

## Discussion

This study reports that the incidence of MSSA-colonization in patients undergoing TJA in South Africa was 31.9%. Analysis of the TJA population indicated a 1.7-fold increased risk for MSSA-colonization in those awaiting TKA compared to THA respectively (OR TKA = 0.58, OR THA = 0.33). In the USA, Ramos et al. found that the prevalence of *S. aureus* colonization in 13,828 consecutive THA, TKA, and spinal fusions was 18.21% [27]. A similar increased prevalence for MRSA was observed in TKA patients (16.3%) compared to THA patients (12.99%) respectively [27].

While no previous studies conducted on *S. aureus* prevalence in a TJA population in South Africa, an epidemiological study found that nosocomial incidence of MSSA and MRSA varied from 1.9 to 3.7 cases per 1000 admissions and 0.03 to 0.08 cases per 1000 admissions, respectively [28]. In the USA, several studies of patients for TJA report the incidence of *S. aureus* colonization to range between 17.5–25.1% for MSSA and 1.8–5% for MRSA [29–31]. There have been several published *S. aureus* prevalence studies in the USA for a TJA population; however, respective data for other regions is limited. Hadi et al. reported that 20.8% of 226 patients awaiting TJA in Arak, Iran, were *S. aureus* carriers [32]. Tsang et al. reported the prevalence for MSSA at 37.9% and MRSA at 2.3%, in 273 TJA patients in the UK [33]. Barbero Allende et al. identified 26.7% of patients colonized with *S. aureus* awaiting TJA in Spain [34].

Several risk factors have been described for *S. aureus* colonization including gender, age, recent hospitalization, ethnicity, genetic predisposition, diabetes mellitus, HIV, hemodialysis, other concurrent skin infections, and antibiotic treatment misuse [35]. This study did not identify any statistically significant risk factors that predicted for the pre-operative colonization with *S. aureus*, which was likely due to the small sample size.

The small cohort of patients showed a trend of increased prevalence of *S. aureus* colonization in HIV positive patients (*p* = 0065). Higher rates of *S. aureus* colonization have been reported in HIV-infected patients with the incidence of MRSA reported as high as 31% [36, 37]. Among nosocomial *S. aureus* infections in South Africa, the overall prevalence of HIV was found to be 32%, which subdivided into 42% and 27% for MRSA and MSSA cases respectively (*p* = 0.106) [28]. In a review of 404 HIV-infected outpatients from two separate centers in Botswana, the nasal colonization of *S. aureus* and MRSA was 36.9% and 3.2% respectively [37]. In HIV-infected patients, those living with children and in high population-density areas were at significantly increased risk for *S. aureus* colonization [37]. All HIV-infected patients in this study were on HAART pre-operatively. The VL of all patients was undetectable before surgery. The low incidence of HIV-infected patients colonized with *S. aureus* may be as a result of good disease control although the population sample was too small to correlate statistically significant conclusions.

In our study, 81.6% of identified *MSSA*-carriers had positive cultures from swabs taken from the anterior nares. The anterior nares are the primary site of *S. aureus* colonization while other areas that may serve as reservoirs include the oropharynx, axillae, groin, perineum, forehead, and neck [12, 38]. Sampling these sites in addition to the anterior nares may improve the detection rates of *S. aureus* however this will infer an increased financial burden.

The decolonization rate using mupirocin and chlorhexidine was 94.74% in this study and is in keeping with literature. Moroski et al. evaluated the effectiveness of a MSSA and MRSA decolonization regime of nasal carriers in patients undergoing primary and revision TJA [26]. The decolonization strategy was a 5-day pre-operative course of intranasal mupirocin only and chlorhexidine was not included [26]. Moroski et al. demonstrated that 34% of MSSA carriers remained colonized despite treatment, while 92% of MRSA-carriers were successfully decolonized [26]. Conversely, Jeans et al. showed that eradication therapy with mupirocin ointment in patients awaiting TJA was most efficacious in patients colonized with MSSA rather than MRSA [39]. Barbero Allende et al. showed that the identical strategy to the one employed in our study resulted in a 98% decolonization rate [34]. The use of intranasal mupirocin and full body chlorhexidine wash yielded good results and supports the combination of both treatment modalities.

A number of strategies regarding the management of potential *S. aureus* colonization of patients awaiting THA and TKA exist. The two most common are a “Screen and Treat” protocol [40, 41] and a policy of universal decolonization [11]. The efficacy of screening and subsequently treating patients colonized with *S. aureus* has been proven [9, 11, 42–44]. However, decolonizing all patients pre-operatively without screening for *S. aureus* is simpler, less time consuming and less dependant on other resources, such as laboratories and staff to process the tests for instance [11]. Universal treatment has also been shown to be more cost-effective [11]. However, the evolution of bacterial resistance to the topical antimicrobials such as mupirocin becomes a factor of concern [45]. The Proceedings of the International Consensus on Orthopaedic Infections could not make any definitive recommendations regarding the most effective approach to managing and treating *S. aureus* colonization pre-operatively due to inconsistent literature reports [11]. Screening and treating or only treating patients with certain medical or demographic risk profiles was, however, strongly discouraged [11]. Risk factors for *S. aureus* colonization vary greatly and are poorly defined. The most suitable approach may actually differ at various individual institutions and be influenced by that institutions’ PJI rate and patient subpopulations seen and treated [11].

There were no post-operative septic sequelae, with no incidences of SSIs or PJIs and no cases of revision TJA in this study at a mean follow-up of 37.34 months. However, this study included a small sample size and this data cannot be extrapolated to be of statistical significance. *S. aureus* colonization has been identified as a modifiable risk for SSI [21]. Patients colonized with *S. aureus* have a nine- to ten-fold increased risk of developing a SSI [10, 16]. MRSA colonization infers an additional four-fold increased risk of infective complications in comparison with MSSA colonization [16]. Molecular typing has helped link *S. aureus* colonization with SSI in THA, TKA, and spine surgery [27]. The exact *S. aureus* subtype found pre-operatively on nasal swab sampling was found to be the infective organism in 85.71% of cases [27].

Ramos et al. found that *S. aureus* colonization was identified as a significant risk factor for SSI in TKA patients [27]. The incidence of SSI in the colonized patients after receiving eradication therapy was lower (2.39%) than in non-colonized patients (4.35%) [27]. Sporer et al. reported that the decolonization of *S. aureus* carriers revealed a 69% reduction in the rate of SSIs in comparison with a control group who had not been screened and treated [29]. In a systematic review of 79 papers from both the Portuguese and English literature, Sadigursky et al. reported that the use of prophylaxis to promote pre-operative decolonization of MRSA decreased the incidence of SSI by almost 39% [38]. Jeans et al. showed that the adoption of an *S. aureus* screening and decolonization program in patients awaiting TJA reduced the PJI rate significantly from 1.92 to 1.41% [39]. Ultimately, £1893 was saved per case by decreasing the PJI rate [39]. Similarly, Barbero Allende et al. showed that the identical decolonization strategy to the one employed in our study resulted in an amelioration of the rate of SSI by 40.7% [34].

There were several major weaknesses identified in the evaluation of the study. The primary limitation was the small sample size of the population. The sample size was calculated in order to evaluate an accurate prevalence denomination for a specific population; however, it was not large enough to determine the secondary aims such as significant risk factors or post-operative consequences.

Secondly, the success of eradication was only assessed on repeat swabs taken immediately after completion of the eradication therapy. A systematic review reported that the successful eradication of *S. aureus* was 95% after 1 week; however, the rate decreased to 64% upon a subsequent review after 2 weeks [46]. Treatment failure risks were associated with colonization at multiple anatomic sites, longer hospital stays, and bacterial resistance to mupirocin [45].

Additionally, the testing for *S. aureus* did not make use of other techniques like polymerase chain reaction (PCR) tests or pre-plating the broth enrichment of swabs for culture to optimize the yield. These have been shown to definitively improve the detection rate of nasal MSSA colonization [33]. Tsang et al. showed that almost one third of both MRSA and MSSA carriers are missed should PCR and pre-plating of the broth enrichment of culture swabs not be added to traditional swabbing techniques [33]. However, the use of PCR testing would have increased the costs of conducting this study and is notable in the context of a developing country such as South Africa. Lastly, only patients awaiting TJA that were screened for *S. aureus* colonization pre-operatively were included in the study, and there was no control group with which to compare all outcomes.

## Conclusion

To our knowledge, this is the first study conducted in Sub-Saharan Africa to evaluate the prevalence of *S. aureus* for patients awaiting TJA. The rate of *S. aureus* colonization in patients undergoing elective TJA in a developing country was 31.9% and is equivalent to reported rates in developed countries. A larger cohort of patients is recommended to determine risk factors and post-operative septic sequelae in this population group.

## Data Availability

The datasets used and/or analyzed during the current study are available from the corresponding author on reasonable request.

## Abbreviations

BMI: Body mass index
CD4+: CD4+ T cell count
HIV: Human immunodeficiency virus
MRSA: Methicillin-resistant *Staphylococcus aureus*
MSSA: Methicillin-sensitive *Staphylococcus aureus*
*S. aureus*: *Staphylococcus aureus*
SSI: Surgical site infection
PJI: Peri-prosthetic joint infection
THA: Total hip arthroplasty
TJA: Total joint arthroplasty
TKA: Total knee arthroplasty
UK: United Kingdom
USA: United States of America
VL: Viral load

## References

[1] Organisation for Economic Co-operation and Development (OECD) (2017), Health at a Glance 2017: OECD Indicators, OECD Publishing, Paris. 10.1787/health_glance-2017-en.

[2] National Joint Registry 16th Annual Report 2019. Published 31 December 2019. NJR Editorial Board. Downloaded from https://reports.njrcentre.org.uk (7 Jan 2020).

[3] American Academy of Orthopaedic Surgeons, American Joint Replacement Registry (AJRR). Fifth AJRR Annual Report on Hip and Knee Arthroplasty Data (2018). Downloaded from http://connect.ajrr.net/2019-ajrr-annual-report (7 Jan 2020).

[4] Kurtz S, Ong K, Lau E, Mowat F, Halpern M (2007). Projections of primary and revision hip and knee arthroplasty in the United States from 2005 to 2030. J Bone Joint Surg Am..

[5] Kurtz SM, Lau EC, Ong KL, Adler EM, Kolisek FR, Manly MT (2017). Which clinical and patient factors influence the national economic burden of hospital readmissions after total joint arthroplasty?. Clin Orthop Relat Res.

[6] Natshara KM, Shelton TJ, Meehan JP, Lum ZC. Mortality during total hip periprosthetic joint infection. J Arthroplasty. 2018 Dec 24, pii: S0883-5403(18)31225-7. Doi: 10.1016/j.arth.2018.12.024. 10.1016/j.arth.2018.12.024 30642705

[7] Beam E, Osmon D. Prosthetic joint infection update. Infect Dis Clin N Am 32 (2018) 843–859. doi: 10.1016/j.idc.2018.06.005. 10.1016/j.idc.2018.06.005 30241717

[8] Bode LG, Kluytmans JA, Wertheim HF, Bogaers D, Vandenbroucke-Grauls CM, Roosendaal R (2010). Preventing surgical-site infections in nasal carriers of Staphylococcus aureus. N Engl J Med.

[9] Sousa RJ, Barreira PM, Leite PT, Santos AC, Ramos MH, Oliveira AF. Preoperative Staphylococcus aureus screening/decolonization protocol before total joint arthroplasty-results of a small prospective randomized trial. J Arthroplasty 2016;31:234e9. 10.1016/j.arth.2015.08.003. 10.1016/j.arth.2015.08.003 26362785

[10] Kalmeijer MD, Coertjens H, van Nieuwland-Bollen PM, Bogaers-Hofman D, de Baere GA, Stuurman A, et al. Surgical site infections in orthopedic surgery: the effect of mupirocin nasal ointment in a double-blind, randomized, placebo-controlled study. Clin Infect Dis 2002;35:353e8. 10.1086/341025. 10.1086/341025 12145715

[11] Åkesson P, Chen AF, Deirmengian GK, Geary M, Quevedo MS, Sousa R, et al. General assembly, prevention, risk mitigation, local factors: proceedings of international consensus on orthopedic infections. J Arthroplasty 34 (2019) S49eS53. doi: 10.1016/j.arth.2018.09.053. 10.1016/j.arth.2018.09.053 30360980

[12] Weiser MC, Moucha CS (2015). The current state of screening and decolonization for the prevention of Staphylococcus aureus surgical site infection after total hip and knee arthroplasty. J Bone Jt Surg.

[13] Wertheim HF, Melles DC, Vos MC, van Leeuwen W, van Belkum A, Verbrugh HA, et al. The role of nasal carriage in Staphylococcus aureus infections. Lancet Infect Dis. 2005 Dec;5(12):751–62 http://www.ncbi.nlm.nih.gov/pubmed/16310147. 10.1016/S1473-3099(05)70295-4 16310147

[14] Agarwala S, Lad D, Agashe V, Sobti A (2016). Prevalence of MRSA colonization in an adult urban Indian population undergoing orthopaedic surgery. J Clin Orthop Trauma.

[15] Goyal N, Miller A, Tripathi M, Parvizi J. Mathicillin-resistant Staphylococcus aureus (MRSA): colonisation and pre-operative screening. Bone Joint J. 2013 Jan;95-B(1):4–9. Doi: 10.1302/0301-620X.95B1.27973. 10.1302/0301-620X.95B1.27973 23307666

[16] Chen AF, Heyl AE, Xu PZ, Rao N, Klatt BA (2013). Preoperative decolonization effective at reducing staphylococcal colonization in total joint arthroplasty patients. J Arthroplasty..

[17] Rodriguez-Merchan EC (2015). Screening and decolonization of MRSA among joint arthroplasty patients: efficacy, cost-effectiveness and durability. J Acute Dis..

[18] Schaumburg F, Ateba Ngoa U, Kö Sters K, Kö Ck R, Adegnika AA, Kremsner PG (2011). Virulence factors and genotypes of Staphylococcus aureus from infection and carriage in Gabon. Clin Microbiol Infect..

[19] [Internet] No authors listed. UNAIDS factsheet June 2019. https://www.aidsinfo.unaids.org (date last accessed 07 Jan 2020).

[20] Conceição T, Santos Silva I, de Lencastre H, Aires-de-Sousa M (2013). Staphylococcus aureus nasal carriage among patients and health care workers in São Tomé and Príncipe. Microb Drug Resist..

[21] Maoz G, Phillips M, Bosco J, Slover J, Mph AS. Mph II. The Otto Aufranc Award modifiable versus nonmodifiable risk factors for infection after hip arthroplasty. 2015:453–9. 10.1007/s11999-014-3780-x PMC4294894 25024028

[22] Naing L, Winn T, Rusli BN (2006). Practical issues in calculating the sample size for prevalence studies. Archives of Orofacial Sciences..

[23] Deinhardt-Emmer S, Sachse S, Geraci J, Fischer C, Kwetkat A, Dawczynski K (2017). Virulence patterns of Staphylococcus aureus strains from nasopharyngeal colonization. J Hosp Infect.

[24] No authors listed. National Health Laboratory Services (NHLS) Handbook Technical Working Group. Document number GPQ0064, Version 1. Downloaded from https://www.nhls.ac.za/diagnostic-services/type-of-tests (last accessed 07 Jan 2020).

[25] Kerbel YE, Sunkerneni AR, Kirchner GJ, Prodromo JP, Moretti VM. The cost-effectiveness of preoperative Staphylococcus aureus screening and decolonization in total joint arthroplasty. J Arthroplasty. 2018;33(7):S191–S195. 10.1016/j.arth.2018.01.032. 10.1016/j.arth.2018.01.032 29510950

[26] Moroski NM, Woolwine S, Schwarzkopf R. Is preoperative staphylococcal decolonization efficient in total joint arthroplasty. J Arthroplasty. 2015 Mar;30(3):444–6 http://www.ncbi.nlm.nih.gov/pubmed/25453634. 10.1016/j.arth.2014.10.017 25453634

[27] Ramos N, Stachel A, Phillips M, Vigdorchik J, Slover J, Bosco JA (2016). Prior Staphylococcus aureus nasal colonization: a risk factor for surgical site infections following decolonization. J Am Acad Orthop Surg..

[28] Fortuin-de Smidt MC, Singh-Moodley A, Badat R, Quan V, Kularatne R, Nana T (2015). Staphylococcus aureus bacteraemia in Gauteng academic hospitals. South Africa. International Journal of Infectious Diseases.

[29] Sporer SM, Rogers T, Abella L. Methicillin-resistant and methicillin-sensitive Staphylococcus aureus screening and decolonization to reduce surgical site infection in elective total joint arthroplasty. J Arthroplasty. 2016 Sep;31(9):144–147. http://www.ncbi.nlm.nih.gov/pubmed/27387479. 10.1016/j.arth.2016.05.019 27387479

[30] Malcolm TL, Robinson LD, Klika AK, Ramanathan D, Higuera CA, Murray TG (2016). Predictors of Staphylococcus aureus colonization and results after decolonization. Interdiscip Perspect Infect Dis.

[31] Walsh AL, Fields AC, Dieterich JD, Chen DD, Bronson MJ, Moucha CS (2020). Risk factors for Staphylococcus aureus nasal colonization in joint arthroplasty patients. J Arthroplasty..

[32] Hadi H, Jabalameli M, Bagherifard A (2018). Ghaznavi-rad E. Staphylococcus aureus colonization in patients undergoing total hip or knee arthroplasty and cost-..

[33] Tsang STJ, McHugh MP, Guerendiain D, Gwynne PJ, Boyd J, Simpson AHRW, et al. Underestimation of Staphylococcus aureus (MRSA and MSSA) carriage associated with standard culturing techniques. Bone Joint Res. 2018 Jan;7(1):79–84 http://www.ncbi.nlm.nih.gov/pubmed/29330346. 10.1302/2046-3758.71.BJR-2017-0175.R1 PMC5805824 29330346

[34] Barbero Allende JM, Romanyk Cabrera J, Montero Ruiz E, Vallés Purroy A, Melgar Molero V, Agudo López R, et al. Eradication of Staphylococcus aureus in carrier patients undergoing joint arthroplasty. Enferm Infecc Microbiol Clin. 2015 Feb;33(2):95–100 https://linkinghub.elsevier.com/retrieve/pii/S0213005X14001268. 10.1016/j.eimc.2014.03.004 24880651

[35] Sakr A, Brégeon F, Mège JL, Rolain JM, Blin O. Staphylococcus aureus nasal colonization: an update on mechanisms, epidemiology, risk factors, and subsequent infections. Front Microbiol. 2018;9(OCT):1–31. 10.3389/fmicb.2018.02419 PMC6186810 30349525

[36] Hidron AI, Kempker R, Moanna A, Rimland D (2010). Methicillin-resistant Staphylococcus aureus in HIV-infected patients. Infect Drug Resist..

[37] Reid MJA, Steenhoff AP, Mannathoko N, Muthoga C, McHugh E, Brown EL (2017). Staphylococcus aureus nasal colonization among HIV-infected adults in Botswana: prevalence and risk factors. AIDS Care - Psychol Socio-Medical Asp AIDS/HIV..

[38] Sadigursky D, Santos H, Américo S, Rios C, Borja L, Filho R (2017). Prophylaxis with nasal decolonization in patients submitted to total knee and hip arthroplasty: systematic review and meta-analysis. Rev Bras Ortop..

[39] Jeans E, Holleyman R, Tate D, Reed M, Malviya A. Methicillin sensitive staphylococcus aureus screening and decolonisation in elective hip and knee arthroplasty. J Infect. 2018;0:1–5. 10.1016/j.jinf.2018.05.012. 10.1016/j.jinf.2018.05.012 29932962

[40] Campbell KA, Cunningham C, Hasan S, Hutzler L, Bosco JA. Risk factors for developing staphylococcus aureus nasal colonization in spine and arthro-plasty surgery. Bull Hosp Jt Dis (2013) 2015;73:276e81. 26630471

[41] Murphy E, Spencer SJ, Young D, Jones B, Blyth MJ (2011). MRSA colonisation and subsequent risk of infection despite effective eradication in orthopaedic elective surgery. J Bone Joint Surg Br.

[42] Schweizer ML, Chiang HY, Septimus E, Moody J, Braun B, Hafner J (2015). Association of a bundled intervention with surgical site infections among patients undergoing cardiac, hip or knee surgery. JAMA.

[43] Kohler P, Bregenzer-Witteck A, Rettenmund G, Otterbech S, Schlegel M (2013). MRSA decolonization: success rate, risk factors for failure and optimal duration of follow- up. Infection.

[44] Sai N, Laurent C, Strale H, Denis O, Byl B. Efficacy of the decolonization of methicillin-resistant Staphylococcus aureus carriers in clinical practice. Anti-microb Resist Infect Control 2015;4:56. 10.1186/s13756-015-0096-x.. 10.1186/s13756-015-0096-x PMC4683972 26688720

[45] Hetem DJ, Bootsma MC, Bonten MJ (2016). Prevention of surgical site infections: decontamination with mupirocin based on preoperative screening for Staphylococcus aureus carriers or universal decontamination?. Clin Infect Dis.

[46] Ammerlaan HSM, Kluytmans JAJW, Wertheim HFL, Nouwen JL, Bonten MJM. Eradication of methicillin-resistant Staphylococcus aureus carriage: a systematic review. Clin Infect Dis. 2009 Apr 1;48(7):922–30 http://www.ncbi.nlm.nih.gov/pubmed/19231978. 10.1086/597291 19231978

